# Physical activity level at work and risk of chronic low back pain: A follow-up in the Nord-Trøndelag Health Study

**DOI:** 10.1371/journal.pone.0175086

**Published:** 2017-04-10

**Authors:** Ingrid Heuch, Ivar Heuch, Knut Hagen, John-Anker Zwart

**Affiliations:** 1 Department of Neurology and FORMI, Oslo University Hospital, Oslo, Norway; 2 Department of Mathematics, University of Bergen, Bergen, Norway; 3 Department of Neuroscience, Norwegian University of Science and Technology, Trondheim, Norway; 4 Norwegian National Headache Centre, Department of Neurology, St. Olavs Hospital, Trondheim, Norway; 5 Faculty of Medicine, University of Oslo, Oslo, Norway; McMaster University, CANADA

## Abstract

**Background:**

Physical activity in leisure time seems to reduce the risk of low back pain, but it is not known whether occupational activity, as recorded in a representative working population, produces a higher or lower risk.

**Objective:**

To study associations between physical activity level at work and risk of chronic low back pain.

**Methods:**

Associations were examined in a Norwegian prospective study using data from the HUNT2 and HUNT3 surveys carried out in the whole county of Nord-Trøndelag. Participants were 7580 women and 7335 men who supplied information about physical activity level at work. Levels considered were sedentary work, work involving walking but no heavy lifting, work involving walking and heavy lifting, and particularly strenuous physical work. Nobody in the cohort was affected by chronic low back pain at baseline. After 11 years, participants reported whether they suffered from chronic low back pain. Generalized linear modelling with adjustment for potential confounders was applied to assess associations with risk factors.

**Results:**

In age-adjusted analyses both women and men showed statistically significant associations between physical activity at work and risk of chronic low back pain, suggesting positive relationships. For particularly strenuous physical work the relative risk of chronic low back pain was 1.30 (95% CI: 1.00–1.71) in women and 1.36 (95% CI 1.17–1.59) in men, compared to sedentary work. Women still showed a general association with activity level after adjustment for education, leisure time physical activity, BMI, smoking and occupational category. In men, the higher risk was only maintained for particularly strenuous work.

**Conclusion:**

In this cohort, women had a higher risk of chronic low back pain with work involving walking and heavy lifting or particularly strenuous work, compared to sedentary work. Men participating in particularly strenuous work also experienced a higher risk of chronic low back pain.

## Introduction

Low back pain (LBP) is a common disabling condition, and it is one of the most frequent causes of long-term sick leave and disability in Norway. Among the Norwegian working population, about 32% experience pain in the lower back during any one month [1]. The risk of LBP differs between demographic categories, and associations have been established with occupational and educational factors [2]. Generally, physical activity is considered to be beneficial, but particular strenuous activities may be associated with increased risk of LBP [3]. In studies of physical activity at work, specific types of work-related exposure seem to provide a higher risk of back pain [3, 4], although it is not clear that there is any definite causal relationship [5]. Occupational and leisure time physical activity may in general have opposing effects on health [6]. In the Global Burden of Disease 2010 study, a major part of the disability due to LBP was found to be related to conditions in the occupational situation [7].

There are numerous studies of risk of LBP in relation to physical activity in specific occupations, as for instance heavy lifting and manual handling among nurses [8], but in most cases these studies do not include a large number of participants. Larger studies based on population data have been performed, however, with a crude classification of the data on physical work activities. Such prospective studies showing greater risk of back pain for physically demanding work have in some cases been based either on disability data of back pain [9], or on population data with a crude characterization of back pain [10]. Smaller prospective studies with crude classification of occupational activity have also indicated higher risk in individuals subject to a heavy physical workload [11–14]. To examine the association between level of activity and risk of LBP, large population-based studies with a prospective design covering many years are still needed.

The purpose of the present study was to explore the associations between levels of physical activity at work and risk of chronic LBP in a large prospective study among the working population. Data were collected in a Norwegian county, and none of the participants included in the analysis were suffering from chronic LBP at baseline. Adjustments were carried out for other factors related to physical activity at work such as overweight, obesity and activity in leisure time, and for background factors as age and education. The same data set has previously been used to study associations between risk of chronic LBP and physical activity in leisure time [15] and BMI [16] and several measures of body size [17].

## Materials and methods

### Study design

The present work is based on information from the Norwegian community-based Nord-Trøndelag Health Study (HUNT). An eleven year prospective study has been conducted considering data from the second survey, HUNT2 (1995–1997) [18] combined with follow-up data from the third survey, HUNT3 (2006–2008) [19].

The whole population in the Nord-Trøndelag county aged 20 years and above were invited to participate in the HUNT2 survey. Each person was asked to fill in a questionnaire on health status, which also included details concerning musculoskeletal conditions. The participants gave information about physical activity at work, education, activity in leisure time, smoking and occupation. Everybody was also invited to a clinical consultation, which included measurements of height and weight. Eleven years later similar information from a questionnaire and a clinical examination was collected in the HUNT3 survey. In the present study baseline data from HUNT2 were considered in combination with information from HUNT3 on health status. Since relevant information was not available in relatively large parts of the youngest and oldest age groups, the study was restricted to those aged 30–69 years when they participated in HUNT2. Information about residence status was supplied by national registries and linked by use of the unique Norwegian personal identification numbers.

One question in the HUNT2 and HUNT3 questionnaires was formulated in this way: “During the last year, have you suffered from pain and/or stiffness in your muscles and joints that has lasted for at least 3 consecutive months?ˮ If a participant answered yes, he or she was given the following question: “Where did you have these complaints?” A number of prespecified regions were listed which could be selected. Respondents who answered yes to the first question and then selected the lower back as a relevant region were regarded in the present study as suffering from chronic LBP [20].

Among 28 906 women and 30 022 men in the age range 30–69 years in the baseline population, 20 272 women and 19 307 men indicated whether they suffered from chronic LBP and gave information about work status, physical activity at work, education, physical activity in leisure time and smoking. This corresponds to a participation rate of 67.2 percent. Altogether 8173 persons, 5072 women and 3101 men, were excluded because they were not employed or did not carry out professional work.

The follow-up study included 24 280 participants in the working population without chronic LBP at baseline, with information about physical activity at work, education, physical activity in leisure time, smoking and BMI. During follow-up 701 persons in this cohort died, 876 persons left the county, 7787 persons living in the county did not participate at end of follow-up or did not give information about chronic LBP and one person disappeared. Totally, 14 915 participants, 7580 women and 7335 men were available for analysis after follow-up, representing 65.7% of the remaining individuals resident in the county and 61.4% of the original cohort.

Each participant in the HUNT2 and HUNT3 surveys signed a written informed consent regarding the collection and use of data for research purposes. The work was approved by the Regional Committee for Medical and Health Research Ethics in Central Norway, and HUNT was also approved by the Norwegian Data Inspectorate.

### Physical activity at work

The participants were asked to indicate the baseline level of physical activity at work in four categories [21]. The categories presented in the questionnaire with illustrative examples were substantially sedentary work (e.g., assembly or desk work), work involving walking, but no heavy lifting (e.g., light manufacturing, salespeople or teachers), work involving both walking and heavy lifting (e.g., postmen, nurses or construction workers) and particularly strenuous physical work (e.g., people involved in heavy agricultural or forestry work and heavy construction work). The information about physical activity collected in HUNT2 was assessed by a reliability and validity study of a subsample [21]. Reliability of the question about work activity was very good, and the information seemed to reflect total energy expenditure and time spent in moderate activity levels.

### Covariates

The participants provided information about gainful employment. This information was for the present study categorized into broad occupational groups with relatively similar work tasks. The first category, referred to as non-professional occupations, included work in shops, offices and public services. The second category, called labourers, included unskilled, semi-skilled and skilled workers, craftsmen and foremen. The third category, lower professional occupations, included nurses, technicians or teachers. The fourth category, farming or forestry, included farmers and forest owners. The fifth category included remaining occupations, such as management positions, drivers, fishermen, self-employed professionals and self-employed businesspersons. The sixth occupational category included participants who did not give any information about specific occupations.

A section of the questionnaire used in HUNT2 dealt with physical activity in leisure time during the last year, including moving to and from work. One question was restricted to light activity, and another referred to hard physical activity, causing sweating or shortness of breath. In the present study, three categories of physical activity in leisure time were considered. The first category represented those who reported no physical activity or light activity only, the second group represented those who reported ≤2 hours hard physical activity per week and the third category represented those who practised ≥3 hours hard physical activity in leisure time per week. In the reliability and validity study [21], the question on hard activity in leisure time showed acceptable repeatability and represented a reasonably valid measure of vigorous activities.

Baseline age was categorized into 10-year intervals in statistical analyses. Education was grouped according to duration as ≤9, 10–12, or ≥13 years. For most participants the first category represents having attended primary and lower secondary school, the second category corresponds to higher secondary school, and the third corresponds to higher education. BMI, defined as weight/height^2^ and computed in kg/m^2^, was subdivided into three groups: <25, 25–29.9, ≥30. Categories of cigarette smoking represented current daily smoking, previous daily smoking and never daily smoking.

### Statistical analysis

Associations between baseline level of physical activity at work and risk of chronic LBP at end of follow-up were assessed by generalized linear modelling for binomial data with a log-link, including adjustment for potential confounders. Physical activity at work was considered both as a categorical and as a continuous variable with the categories given scores, 1 for sedentary work, 2 for work involving walking and no heavy lifting, 3 for work involving walking and heavy lifting and 4 for particularly strenuous work. A test for deviation from linearity in work activity as a continuous variable was performed by testing whether inclusion of an additional squared term of the activity score provided a significantly better fit in the generalized linear model. All analyses were carried out separately for women and men.

Initial analyses incorporated adjustment for age only, and then additional adjustment was introduced for other potential risk factors as physical activity in leisure time [15], BMI [22] and smoking [23]. Finally, adjustment was carried out for education, known to be a risk factor for LBP [24], and occupational categories [25]. All variables adjusted for were regarded as categorical. Separate tests were performed for interaction between occupational physical activity as a continuous variable and each adjustment variable. All statistical analyses were carried out using IBM SPSS version 21 (IBM Corp., Armonk, New York).

## Results

A substantial number of women and men belonged to each group of physical activity at work, although few women practised particularly strenuous physical work (Table 1). Among women, the most common activity category represented work involving walking but no heavy lifting, while in men sedentary work was the most frequent category. Physical activity at work was related to several other risk factors in the baseline population. Among the participants with education ≥13 years, few were engaged in particularly strenuous work, and the majority of the men were occupied in sedentary work (Table 1). Among the current smokers, a larger proportion practised work involving walking and heavy lifting than in the other smoking categories, especially among women. Non-professional workers had a high proportion of sedentary work. Farmers had a very high proportion of particularly strenuous physical work. Only moderate differences in the percentages of physical activity at work were observed between categories of age, leisure time physical activity and BMI.

**Table 1:** Distribution of physical activity at work in subgroups of other risk factors, among participants without chronic LBP at baseline included in statistical analyses.

	Women					Men				
	Physical activity at work					Physical activity at work				
		Sedentary work	Work involving walking, no heavy lifting	Work involving walking and heavy lifting	Particularly strenuous physical work		Sedentary work	Work involving walking, no heavy lifting	Work involving walking and heavy lifting	Particularly strenuous physical work
	Total	%	%	%	%	Total	%	%	%	%
Complete data set	7580	28.9	38.2	30.1	2.8	7335	35.3	25.5	18.7	20.5
**Age (years)**										
30–49	5131	30.0	35.8	31.9	2.3	4612	35.0	24.5	20.4	20.0
50–69	2449	26.6	43.3	26.3	3.8	2723	35.8	27.1	15.8	21.2
**Leisure time physical activity**										
Light only	3763	27.4	38.3	31.5	2.9	2698	32.4	25.3	20.2	22.1
**≤** 2 hours hard per week	3372	31.0	38.4	28.2	2.4	3635	38.0	26.2	18.8	17.0
**≥** 3 hours hard per week	445	26.3	36.0	32.6	5.2	1002	33.3	23.5	14.4	28.8
**Education (years)**										
**≤** 9	1924	14.5	45.0	35.4	5.1	1589	20.6	24.9	23.6	30.9
10–12	3485	36.0	29.8	31.4	2.8	3686	26.9	24.1	24.1	25.0
**≥** 13	2171	30.3	45.7	23.1	0.8	2060	61.7	28.5	5.4	4.3
**BMI (kg/m2)**										
< 25	3859	31.1	37.8	29.0	2.1	2419	32.9	27.5	18.4	21.2
25–29.9	2802	26.7	39.2	30.8	3.2	4009	36.8	24.7	18.8	19.7
**≥** 30	919	26.2	37.0	32.2	4.6	907	35.4	23.3	19.3	22.1
**Cigarette smoking**										
Never	3571	29.1	40.2	27.5	3.1	3296	36.8	24.2	16.9	22.1
Former	1946	31.2	35.9	30.1	2.7	2371	36.0	26.7	19.1	18.1
Current	2063	26.4	36.8	34.5	2.4	1668	31.4	26.3	21.8	20.6
**Occupational categories**										
Non-professional	2304	53.2	30.6	15.8	0.4	540	56.7	26.7	13.5	3.1
Labourers	1377	6.9	43.7	46.7	2.7	2119	13.7	32.7	34.7	18.8
Lower professional	1858	16.5	46.9	36.1	0.5	981	46.3	46.2	5.2	2.3
Farming or forestry	387	1.8	25.8	41.3	31.0	978	2.4	3.3	15.4	78.9
Other occupations	577	51.8	35.0	12.1	1.0	1806	65.5	18.3	9.9	6.3
Not specified	1077	24.0	38.6	34.5	2.8	911	36.6	23.8	20.2	19.4

LBP, low back pain.

In women, the percentage of chronic LBP at end of follow-up increased with increasing levels of baseline physical activity at work (Table 2), although the percentages of women with chronic LBP were similar in the group involving walking and heavy lifting and the group with particularly strenuous physical work. In men, the percentage of chronic LBP at end of follow-up increased consistently with increasing levels of baseline occupational physical activity (Table 2).

**Table 2:** Percentage of chronic LBP at end of follow-up by physical activity at work, among participants without chronic LBP at baseline included in statistical analyses.

	Women		Men	
	Number	With chronic LBP at end of follow-up (%)	Number	With chronic LBP at end of follow-up (%)
**Physical activity at work**				
Sedentary work	2192	373 (17.0)	2590	319 (12.3)
Work involving walking and no heavy lifting	2895	557 (19.2)	1869	236 (12.6)
Work involving walking and heavy lifting	2280	504 (22.1)	1374	216 (15.7)
Particularly strenuous physical work	213	47 (22.1)	1502	249 (16.6)
Total	7580	1481 (19.5)	7335	1020 (13.9)

LBP, low back pain.

In age-adjusted analyses of risk of chronic LBP, both women and men showed significant increasing relationships with level of physical activity at work (Tables 3 and 4), with about 30% higher risk in the categories involving walking and heavy lifting and particularly strenuous work compared to sedentary work. Further adjustment for physical activity in leisure time, BMI and smoking produced only slightly weaker relationships. In women, additional adjustment, first for education and then for occupational category, again led to slightly weaker associations (Table 3). In men, however, the association to a large extent disappeared after adjustment for education and occupational category, except for those engaged in particularly strenuous physical work, who still showed a higher risk than men occupied in sedentary work (Table 4). Additional squared terms in the score for work activity did not provide significant contributions (results not shown).

**Table 3:** Associations between physical activity at work and risk of chronic LBP in women without chronic LBP at baseline.

	Adjustment for age	Additional adjustment for leisure time activity, BMI and smoking^a^	Additional adjustment for education^b^	Additional adjustment for occupational category^c^
Physical activity at work	RR (95% CI)	RR (95% CI)	RR (95% CI)	RR (95% CI)
Sedentary work	1.00 (reference)	1.00 (reference)	1.00 (reference)	1.00 (reference)
Work involving walking, no heavy lifting	1.13 (1.00–1.27)^d^	1.12 (1.00–1.26)^e^	1.14 (1.01–1.29)	1.11 (0.97–1.26)
Work involving walking and heavy lifting	1.31 (1.16–1.48)	1.27 (1.13–1.43)	1.26 (1.12–1.43)	1.21 (1.06–1.38)
Particularly strenuous physical work	1.30 (1.00–1.71)^e^	1.27 (0.97–1.66)	1.24 (0.95–1.63)	1.24 (0.92–1.67)
*P*, categorical effect	<0.001	0.001	0.002	0.049
*P*, linear effect^f^	<0.001	<0.001	<0.001	0.006

LBP, low back pain; RR, relative risk; CI, confidence interval.

^a ^Adjustment for age, leisure time activity, BMI and smoking.

^b ^Adjustment for age, leisure time activity, BMI, smoking and education.

^c ^Adjustment for age, leisure time activity, BMI, smoking, education and occupational category.

^d ^CI does not include the exact value 1.0.

^e ^CI includes the exact value 1.0.

^f ^For linear effect of score for physical activity at work.

**Table 4:** Associations between physical activity at work and risk of chronic LBP in men without chronic LBP at baseline.

	Adjustment for age	Additional adjustment for leisure time activity, BMI and smoking^a^	Additional adjustment for education^b^	Additional adjustment for occupational category^c^
Physical activity at work	RR (95% CI)	RR (95% CI)	RR (95% CI)	RR (95% CI)
Sedentary work	1.00 (reference)	1.00 (reference)	1.00 (reference)	1.00 (reference)
Work involving walking, no heavy lifting	1.03 (0.88–1.21)	1.03 (0.88–1.21)	0.96 (0.82–1.13)	0.96 (0.82–1.13)
Work involving walking and heavy lifting	1.28 (1.09–1.50)	1.24 (1.06–1.46)	1.08 (0.91–1.27)	1.08 (0.90–1.29)
Particularly strenuous physical work	1.36 (1.17–1.59)	1.35 (1.16–1.58)	1.16 (0.98–1.36)	1.22 (1.01–1.49)
*P*, categorical effect	<0.001	<0.001	0.17	0.099
*P*, linear effect^d^	<0.001	<0.001	0.05	0.041

LBP, low back pain; RR, relative risk; CI, confidence interval.

^a ^Adjustment for age, leisure time activity, BMI and smoking.

^b ^Adjustment for age, leisure time activity, BMI, smoking and education.

^c ^Adjustment for age, leisure time activity, BMI, smoking, education and occupational category.

^d ^For linear effect of score for physical activity at work.

Analyses of risk of chronic LBP by combinations of education and physical activity at work were conducted among women (Table 5) and men (Table 6). As few women were involved in particularly strenuous physical work, this category was combined with the category representing walking and heavy lifting in these analyses. Increasing risk estimates with heavier physical activity at work were observed among women with ≤ 9 and 10–12 years of education, but not among those with ≥ 13 years of education (Table 5). Among men, those with particularly strenuous work had the highest risk estimates in all educational categories (Table 6).

**Table 5:** Risk of chronic LBP by combinations of education and physical activity at work, in women without chronic LBP at baseline.

	Duration of education (years)		
	≤ 9	10–12	≥ 13
Physical activity at work	RR (95% CI)^a^	RR (95% CI)^a^	RR (95% CI)^a^
Sedentary work	1.00 (reference)	0.89 (0.68–1.17)	0.88 (0.65–1.20)
Work involving walking, no heavy lifting	1.07 (0.82–1.42)	1.11 (0.85–1.47)	0.78 (0.57–1.06)
Work involving walking and heavy lifting, or particularly strenuous physical work	1.14 (0.86–1.51)	1.23 (0.94–1.62)	0.88 (0.63–1.23)

LBP, low back pain; RR, relative risk; CI, confidence interval.

^a^Adjustment for age and occupational category.

**Table 6:** Risk of chronic LBP by combinations of education and physical activity at work, in men without chronic LBP at baseline.

	Duration of education (years)		
	≤ 9	10–12	≥ 13
Physical activity at work	RR (95% CI)^a^	RR (95% CI)^a^	RR (95% CI)^a^
Sedentary work	1.00 (reference)	0.92 (0.69–1.23)	0.58 (0.43–0.79)
Work involving walking, no heavy lifting	0.95 (0.67–1.34)	0.89 (0.66–1.20)	0.51 (0.35–0.75)
Work involving walking and heavy lifting	0.97 (0.68–1.37)	1.00 (0.74–1.34)	0.83 (0.49–1.43)
Particularly strenuous physical work	1.17 (0.84–1.63)	1.10 (0.80–1.50)	0.99 (0.56–1.75)

LBP, low back pain; RR, relative risk; CI, confidence interval.

^a ^Adjustment for age and occupational category.

None of the other risk factors considered showed significant interaction with level of work activity. In particular, no interaction was observed with physical activity in leisure time (*P* = 0.46 in women and *P* = 0.27 in men), with education (*P* = 0.10 in women and *P* = 0.67 in men) or with occupational group (*P* = 0.68 in women and *P* = 0.28 in men). No significant differences were seen in the effect of level of work activity between women and men (*P* = 0.85).

To assess the representativeness of the data set analysed, percentages were computed among the 9365 individuals included in the original cohort with no data available at the end of follow-up. This group had a lower proportion of women than those analysed (41% vs. 51%) and a higher proportion of young people in the age interval 30–49 years (72% vs. 65%). However, the proportions of physical activity at work were quite similar (31% vs. 32% sedentary work, 30% vs. 32% work involving walking but no heavy lifting, 25% vs. 24% work involving both walking and heavy lifting, and 13% vs. 11% of particularly strenuous work).

## Discussion

This study showed a significant association between level of physical activity at work and risk of chronic LBP in women, even after adjustment for other important risk factors. In particular, the large group of women engaged in work involving walking and heavy lifting appeared to have an increased risk. In men, an increased risk could be established in those practising particularly strenuous work.

A strength of our study is the large population-based data set, with a great majority of the individuals belonging to a uniform ethnic group [18]. The condition studied is restricted to chronic pain in the lower back, producing a more specific classification than in several other studies. The cohort was defined in such a way that none of the participants suffered from chronic LBP at baseline. With a long period between recording of baseline information and possible classification of chronic LBP in a prospective study design, it is unlikely that initial stages of the condition should influence the physical activities reported. Participation was far from complete at end of follow-up, but although the proportion lost in the cohort depended on sex and age, it was essentially independent of physical activity level at work.

In this study, as in many other cases, only self-reported information about LBP was available. Pain intensity was not recorded and pain status in the 11 year period between the HUNT2 and HUNT3 surveys was unknown. Moreover, no information was available on changes in occupational physical activity in the intervening period. Work status recorded in HUNT3 has not been taken into account as that may be influenced by LBP status at end of follow-up.

The classification of self-reported physical activity at work has its limitations and is not very detailed. The general classification of occupational physical activity into four groups is quite similar to that described by Saltin & Grimby in 1968 [26], but differs especially in the use of occupational examples provided to the respondents and in the particular references to walking and lifting. In a systematic review comparing repeatability and validity of different questionnaires assessing occupational physical activity, the information from HUNT2 [21] was regarded to have good repeatability and moderate construct validity [27].

As physical activity at work may be strongly related to other risk factors for back pain, it is important to carry out adjustment for relevant potential confounders. In our study information was available on several such confounders, but adjustment for leisure time activity, BMI and smoking did not substantially affect the association with work activity. In contrast, adjustment for education weakened the association considerably in men. Previous work in the Nord-Trøndelag population [28] showed that education has a strong independent effect on disability from back pain which is not mediated by working conditions or occupational class. The categorization based on duration of education may also be an indicator of socioeconomic status [28] and thus adjustment for education is essential.

The purpose of adjusting for occupational group is to obtain more comparable levels of work activity, but the occupational categories included in this study were crude and may have combined rather different occupations. Control for occupation may lead to overadjustment if there are large differences in physical activity between different occupational groups. In our data for men, however, with adjustment already carried out for education, there was no indication that additional adjustment for occupational category led to a weaker relationship. In women, the final risk estimates may have been subject to some overadjustment, and thus the true relationship with work activity may be slightly stronger than indicated by the fully adjusted values. To avoid potential further overadjustment, no attempt was made to take psychosocial factors at work into account, even though such factors may in principle be associated both with physical activity and risk of LBP [29]. Classification of physical activity at work, performed by the respondents themselves, may also be influenced by other predisposing factors for chronic LBP.

The analyses cross-classified by both education and activity level at work showed a tendency to lower risk estimates of LBP in groups with a long duration of education, but the pattern according to activity level within educational categories was not always consistent. No evidence was found in our analyses that the potential relation with activity level at work differs between categories defined by other factors such as leisure time activity.

Several predisposing factors related to physical activity have been associated with the occurrence of chronic LBP. There has been considerable interest in possible associations between LBP and general activity, both during occupation and in leisure time. Many studies have been carried out, often with small sample size and with a design not adapted to addressing etiologic questions. Despite heterogeneous results, some reviews have found moderate to strong associations with work-related exposure [3, 4], but at least one review found few associations [5].

In our study, we observed a relatively modest increase in risk of chronic LBP associated with a heavy workload. Other studies with crude categorization of occupational workload have partially found stronger associations [9, 12]. One Norwegian study dealt with disability due to back pain [9]. In a Danish study [12], those with heavy work at baseline often changed their occupations, and therefore classification at baseline was not necessarily representative of the entire period of follow-up. As in our study, periods of follow-up were relatively long in these studies, 5 years [12] and 7 years [9]. Positive associations between occupational load and risk of back pain were also found in a Canadian study with 2 years follow-up time [10] and in a Finnish study with 1 year follow-up [11]. Among these studies [9–12], only two [10, 11] dealt with a population free from back pain at baseline, and only two [11, 12] dealt exclusively with LBP.

A few prospective studies, however, have only focused on particular levels of physical activity, as, for example, sedentary work compared to other activities, with few indications of an increased risk [30, 31]. In a Norwegian 3-year study, highly demanding jobs, prolonged standing and awkward lifting appeared as the most important predictors of LBP [32]. A meta-analysis found increased annual incidence of LBP related to intensity and frequency of lifting at work [33]. In an English one-year follow-up study, work involving heavy lifting or standing and walking was associated with LBP [34]. A Danish follow-up study of female employees demonstrated an increase in back pain by lifting and carrying with the back bent forward [35]. This is specific workload information that is not available in studies with broad categorization.

Work activity and leisure time physical activity do not necessarily represent the same strain on the back, and it is not obvious whether physical activity at the workplace and in leisure time should lead to associations in the same direction. At least two studies dealing separately with both kinds of activities have shown inverse associations with leisure time physical activity and positive associations with occupational activities [6, 36]. In other studies, work involving a large amount of bending and twisting has shown associations with LBP [3, 4], indicating that physical activity under special occupational circumstances should be considered separately. In a Danish follow-up of workers without severe pain at baseline, highly repetitive work was associated with LBP [37]. In the current dataset an inverse relationship between leisure time physical activity and risk of chronic LBP was found in both sexes, but practising 3 hours or more of hard physical activity per week did not provide any further decrease in risk of chronic LBP [15].

Although associations did not differ significantly between women and men in our data, a higher risk was suggested for work involving walking and heavy lifting in women, but not in men. It is not clear that the classification according to activity level represents similar underlying loads in women and men, especially as women and men to a large extent perform different tasks. Women and men may also have a different perception of pain [38]. Such differences may be related to biological, psychological and social aspects [39].

There are indications that workload and occupational physical activity can be reliably assessed by questionnaires [12], but in future studies the information on physical activity at work should preferably not be self-reported. Detailed observations will be essential, with objective measures of occupational workload, for example with automatic recording of activities. Duration of occupational exposure should be identified. However, collecting such information in large prospective population-based studies will require substantial resources.

In summary, this study indicates that a heavy physical workload increases the risk of chronic LBP in both women and men, although not necessarily in the same way. Particularly strenuous work seems to carry a higher risk, and so does work for women involving walking and heavy lifting.
